# Insights into the Pharmacogenetics of Tacrolimus Pharmacokinetics and Pharmacodynamics

**DOI:** 10.3390/pharmaceutics14091755

**Published:** 2022-08-23

**Authors:** Mercè Brunet, Marçal Pastor-Anglada

**Affiliations:** 1 Farmacologia i Toxicologia, Servei de Bioquímica i Genètica Molecular, Centre de Diagnòstic Biomèdic. Hospital Clínic de Barcelona, Universitat de Barcelona, 08036 Barcelona, Spain; 2 Institut d’Investigacions Biomèdiques August Pí i Sunyer (IDIBAPS), 08036 Barcelona, Spain; 3 Centro de Investigación Biomédica en Red Enfermedades Hepáticas y Digestivas (CIBEREHD), 28029 Madrid, Spain; 4 Molecular Pharmacology and Experimental Therapeutics (MPET), Departament de Bioquímica i Biomedicina Molecular, Institut de Biomedicina, Universitat de Barcelona (IBUB), 08028 Barcelona, Spain; 5 Institut de Recerca Sant Joan de Déu (IRSJD), 08950 Esplugues de Llobregat, Spain

**Keywords:** tacrolimus, pharmacogenetics, pharmacokinetics, pharmacodynamics, personalized treatment, intrapatient variability, fast metabolizer, membrane transporters

## Abstract

The influence of pharmacogenetics in tacrolimus pharmacokinetics and pharmacodynamics needs further investigation, considering its potential in assisting clinicians to predict the optimal starting dosage and the need for a personalized adjustment of the dose, as well as to identify patients at a high risk of rejection, drug-related adverse effects, or poor outcomes. In the past decade, new pharmacokinetic strategies have been developed to improve personalized tacrolimus treatment. Several studies have shown that patients with tacrolimus doses C_0_/D < 1 ng/mL/mg may demonstrate a greater incidence of drug-related adverse events and infections. In addition, C_0_ tacrolimus intrapatient variability (IPV) has been identified as a potential biomarker to predict poor outcomes related to drug over- and under-exposure. With regard to tacrolimus pharmacodynamics, inconsistent genotype-phenotype relationships have been identified. The aim of this review is to provide a concise summary of currently available data regarding the influence of pharmacogenetics on the clinical outcome of patients with high intrapatient variability and/or a fast metabolizer phenotype. Moreover, the role of membrane transporters in the interindividual variability of responses to tacrolimus is critically discussed from a transporter scientist’s perspective. Indeed, the relationship between transporter polymorphisms and intracellular tacrolimus concentrations will help to elucidate the interplay between the biological mechanisms underlying genetic variations impacting drug concentrations and clinical effects.

## 1. Introduction

Interest in pharmacogenetics has grown significantly in the last decade. However, the strength of evidence required for the clinical implementation of pharmacogenetics is highly debated. Most of the literature on pharmacogenetics has focused on pharmacokinetics as the phenotype of interest, including the implementation of individualized dosing to achieve target drug exposure. Some examples of this approach are the guidelines and consensus documents for dosing tacrolimus, based on the *CYP3A5* genotype [1] and the cluster of *CYP3A5* and *CYP3A4* genotypes, respectively [2,3].

Tacrolimus is the main component in current immunosuppressive therapies in the prophylaxis of allograft rejection [2]. Tacrolimus management is complex, due to its narrow therapeutic index and high interpatient and intrapatient (the latter being associated with specific clinical situations) pharmacokinetic variability, with drug overexposure having been linked to nephrotoxicity, neurotoxicity, diabetes, opportunistic infections, increased cardiovascular risk, and malignancies; conversely, underexposure might result in graft rejection (TCMR or ABMR), SCR, or sustained alloreactivity, with a negative impact on graft function and the ultimate loss of the graft [2,3].

Current therapeutic drug monitoring (TDM) strategies employ individual dose adjustment to achieve a particular target whole blood trough concentration (C_0_), which is associated with drug-related adverse event minimization and acceptable efficacy in preventing rejection, but there are clear limitations to achieving personalized tacrolimus therapy based on the patient’s immune status and their individual response to treatment [4]. Pharmacogenetic biomarkers influencing tacrolimus exposure and response have the potential to enable individualization of the starting dose and aid the achievement of target blood concentrations in the first days after transplantation. Genetic factors including CYP3A5*3, CYP3A4*22, CYP4A4*1B, and *ABCB1* have been reported frequently for their influence on tacrolimus dose requirements [2,3,5]. More recently, several genetic polymorphisms in selected *SLC* and *ABC* transporter encoding genes have been reported to be associated with tacrolimus exposure and its toxicity [6].

Regarding new tacrolimus TDM approaches, high intra-patient variability (IPV) in tacrolimus C_0_ concentrations can be considered a potential biomarker of treatment outcome because the alternation between episodes of overexposure and underexposure may have an impact on a patient’s risk of toxicity or rejection (TCMR, ABMR) [7,8,9,10,11,12,13,14]. Considering that *CYP3A5* expressers require almost double the conventional tacrolimus dose to achieve target concentrations, compared with non-expressers, these patients should be more susceptible to higher IPV. Unfortunately, there is a lack of conclusive studies evaluating a potential direct effect of the *CYP3A5* genotype, or the clusters of *CYP3A5* and *CYP3A4* genotypes, on tacrolimus C_0_ IPV. Numerous studies report that patients with a low C_0_/dose (D) ratio of tacrolimus (C_0_/D ratio < 1.05 ng/mL/mg) are at increased risk of poor outcomes after kidney transplantation [15,16,17], with a significant effect of CYP3A5*3 and drug-drug interactions on tacrolimus C_0_/D [18].

This short review outlines the requirements of therapeutic approaches, considering the pharmacokinetic and pharmacogenetic biomarkers for initial tacrolimus dose election and adjustments to achieve and maintain appropriate tacrolimus concentrations. The influence of the *CYP3A* genotype on tacrolimus IPV and the C_0_/D ratio, the potential role of genetic variants of transporters in IPV, and the influence of other factors determinants of high IPV are discussed below.

## 2. CYP3A Pharmacogenetics and Its Influence on Tacrolimus Pharmacokinetics, IPV, C_0_/D Ratio, and Clinical Outcome

Tacrolimus is metabolized by gastrointestinal and hepatic CYP3A isoenzymes, mainly CYP3A5 and CYP3A4 [19]. Genetic variations in *CYP3A5* and *CYP3A4* have been consistently associated with pharmacokinetic variability in multiple studies and meta-analyses. The main enzyme involved in tacrolimus pharmacokinetics is *CYP3A5*, followed by *CYP3A4* [20]. The main variant of *CYP3A5* is a single-nucleotide polymorphism (SNP) affecting intron 3 of *CYP3A5* (6986A > G; rs776746 SNP). Approximately 80–85% of Caucasian patients are homozygous for this variant CYP3A5*3 allele (CYP3A5 *3/*3), which results in aberrant splicing and the absence of protein and, thus, CYP3A5 activity. Individuals who are carriers of at least one CYP3A5*1 allele are classified as CYP3A5 expressers (CYP3A5 *1/*3; CYP3A5 *1/*1) [21,22,23,24].

Regarding other candidate SNPs that have been studied, an intron 6 C > T SNP (CYP3A4*22, rs35599367 SNP) is linked to CYP3A4 hepatic expression [25] and may partially explain the variability in tacrolimus pharmacokinetics. CYP3A4*22 has been shown to be associated with tacrolimus dose requirements in stable renal [26] and liver transplant recipients [27]. Kidney transplant recipients who carry the CYP3A4*22 variant allele require significantly lower tacrolimus doses than non-carriers [28]. However, evidence supporting tacrolimus dose selection and adjustment, based on the CYP3A4*22 genotype, is less conclusive than the robust evidence available for *CYP3A5*.

Of interest, some studies reported a significant association between CYP3A4 *22 and tacrolimus and cyclosporine pharmacokinetics in kidney transplant recipients [29], although Lunde et al. [30] found a differential impact of the CYP3A4 *22 genetic variant on tacrolimus and cyclosporine pharmacokinetics. Those recipients receiving cyclosporine required only half the conventional dose to reach target concentrations, whereas, in those treated with tacrolimus, no association with the CYP3A4 *22 genotype and C_0_/D was observed. One possible explanation for these observations relies upon the differences in cyclosporine and tacrolimus metabolism. Indeed, cyclosporine appears to be metabolized predominantly by CYP3A4, whereas the main enzyme implicated in tacrolimus biotransformation is CYP3A5 [20,31].

Importantly, the minor allele frequency (MAF) of CYP3A5*3 shows high variability among ethnicities, ranging from 0.18 in African ethnicities to 0.94 in Europeans. This results in significant differences in CYP3A5 expression and activity, with only 15–20% of whites expressing CYP3A5 at a detectable level [5,32]. Thus, ethnicity plays an important role in the real impact of genetic variants on tacrolimus inter-individual variability exposure and therapeutic effect. In this regard, the results reported by Mohamed et al. [33] demonstrate that CYP3A5*3 is an important predictor of tacrolimus exposure in European American, Native American, African American, and Asian American kidney transplant recipients. However, the magnitude of the influence of CYP3A5*3 on tacrolimus pharmacokinetics varied by ancestry, with the greatest effect in the European American population. In addition, in this study, CYP34*22 was only significantly associated with tacrolimus exposure in a population with European ancestry. Within the framework of the 1000 Genomes Project data [32], human populations have been divided into 5 groups: EUR, Europeans; AFR, Africans; AMR, admixed Americans; EAS, East Asians; and SAS, South Asians, supporting the view that the frequency of the CYP3A5*3 allele and other genetic variants might differ notably among these ethnic population subsets. Even more, high diversity in the frequency of the variant alleles has been observed within each ethnic subset [5]. Consequently, it is evident that algorithms and dosing models for tacrolimus need to be developed and validated, taking into account ethnicity-related genetic heterogeneity.

Other genes and SNPs of interest, such as the CYP-mediated drug oxidation POR *28 (rs1057868), and the nuclear receptor, peroxisome proliferator-activated receptor alpha (*PPARA*), have also been investigated. POR *28 (rs1057868) seems to determine an increased POR, leading to enhanced CYP3A5 and CYP3A4 enzyme activity and, accordingly, faster tacrolimus metabolism. Some studies showed an increased CYP3A5-mediated tacrolimus metabolism in kidney recipients, POR *28 carriers, and CYP3A5 non-expressors, who require about a 15–20% dose increase to achieve target concentrations [5]. Regarding the *PPARA* gene, two genetic variants, *PPARA* c.209-1003G > A and c.208+3819A > G, can reduce PPAR-α expression and may partially explain the intra- and inter-individual variability in CYP3A expression and activity. In this regard, some studies in kidney transplant recipients demonstrate that carriers for these variants (mainly c.208+3819A > G) showed increased tacrolimus exposure [2,34], although other research groups have obtained opposite results [22,35]. Therefore, based on the available data, we believe that the effect of these SNPs on tacrolimus systemic exposure and its effects seem to be much less clinically relevant as putative biomarkers than the CYP3A5*3 or the cluster CYP3A5*3/CYP3A4*22 genotypes, at least in the White population [2,5].

Regarding the impact of pharmacogenetics on clinical outcome, several studies showed a lack of association between *CYP3A* and *ABCB1* genetic variants and the risk of acute rejection [2]. Moreover, although tacrolimus-induced nephrotoxicity is likely related to intrarenal drug concentrations, controversial results have been reported when addressing the potential role of the *CYP3A5* and *ABCB1* genotypes in these adverse reactions [5]. Recent results demonstrate that the recipient and donor *CYP3A5* (rs776746) and *ABCB1* (rs1045642) genotypes do not determine allograft tacrolimus exposure and nephrotoxicity in renal transplant recipients. In terms of adjusting for dose, recipients who were CYP3A5 expressors had lower C_0Blood_ values than non-expressors, while delayed graft function was associated with higher C_0Blood_ values. However, tacrolimus dose and C_0Blood_ were significant predictors of C_Graft_, which is associated with acute nephrotoxicity when very high C_Graft_ values (>800 pg/mg tissue) are achieved [36]. More standardized clinical trials are needed to assess the relationship between *CYP3A5* SNP and tacrolimus pharmacodynamics in children, particularly in terms of acute rejection and nephrotoxicity. A systematic analysis of *ABCB1* and *CYP3A* SNPs in children who underwent organ transplantation found a strong link between *CYP3A5* genotype and tacrolimus pharmacokinetics, although no evidence supporting a role for *CYP3A5*′s genetic variants on its pharmacodynamics was provided. On the other hand, most studies have failed to find a link between the *ABCB1* SNP and the pharmacokinetics of tacrolimus in children. However, although available literature is still scarce, there seems to be a link between *ABCB1* SNPs and tacrolimus pharmacodynamics, acute rejection, and nephrotoxicity [37]. Recent studies investigated the non-genetic and genetic risk factors of developing post-transplant diabetes mellitus (PTDM) in renal transplant patients who were receiving tacrolimus [38,39]. Indeed, although, in terms of general trends, genetic background may be a risk factor for PTDM, there is still controversy regarding which genes and SNPs would be involved in this adverse outcome. Zhang et al. [38] demonstrated that in a cohort of Chinese adult renal transplant patients, age (>50 years) and the *CYP24A1* (rs2296241) A allele were independently correlated with the development of PTDM, while no significant differences were observed in pharmacokinetics and *CYP3A5* (rs776746) and PPAR (rs4253728) polymorphisms between recipients with and without PTDM. Observations in a cohort of White pediatric renal transplant patients showed that homozygous carriers of POR*28 (rs1057868) or wild-type *ABCB1* (rs1045642) gene variants were more frequent in PTDM than in control patients. A genetic score based on these variants demonstrated that POR*28/*28 and *ABCB1* CC or CT genotype carriers were at a significantly higher risk of developing PTDM after transplantation. Current results regarding the association between the *CYP3A5* genotype and BP/hypertension have been inconsistent. Xi et al. [40] performed a meta-analysis including ten studies (2799 cases and 6794 controls) to evaluate the association between *CYP3A5* (rs776746) polymorphism and BP/hypertension. Overall, no associations were observed between the rs776746 polymorphism and BP/hypertension. In the subgroup analysis, CYP3A5*1 carriers had lower systolic BP compared with non-carriers in White populations. This meta-analysis suggested a modestly significant association between the *CYP3A5* rs776746 polymorphism and systolic BP in White populations. Given the limited sample size, additional studies are necessary to investigate the role of CYP3A5 in the regulation of BP and the pathogenesis of hypertension. In summary, the available data are controversial and show inconsistencies when assessing the influence of tacrolimus pharmacogenetics in the development of drug-related adverse events. This may be partially explained by ethnicity variability and a small sample size. Therefore, multicentric studies addressing this question are warranted.

Despite the lack of convincing data linking tacrolimus pharmacogenetics and clinical outcome, there is robust evidence regarding the association between the *CYP3A* genotype and tacrolimus pharmacokinetics. Importantly, guidelines for the genotype-based selection of the initial tacrolimus dose have been published by several expert working groups. The Clinical Pharmacogenetics Implementation Consortium (CPIC) [1] recommends increasing the starting dose to 1.5 to 2 times that of the conventional starting dose, with the total starting dose not exceeding 0.3 mg/kg/day, while subsequent dosing should be determined based on TDM. The Dutch Pharmacogenetics Working Group (DPWG) recommends a 1.5- and 2.5-fold increase in the conventional dose for heterozygous and homozygous expressers, respectively, and subsequent dosing should be based on TDM [41]. In addition, the experts of the IATDMCT also provide some recommendations and an executive summary regarding genotype-based tacrolimus dosing [2]; they conclude that while a CYP3A5-based initial tacrolimus dose may facilitate tacrolimus dosing, there is currently no convincing clinical evidence that the use of pharmacogenetic-based tacrolimus dosing improves clinical outcomes after solid organ transplantation.

Some recent studies provide improved tacrolimus PK/PG models and dosing algorithms. Andrews et al. [42] developed a population pharmacokinetic model to predict the individual starting dose of tacrolimus after kidney transplantation. This study demonstrated that multiple clinical (albumin, creatinine, and hematocrit), demographic (age and bodyweight surface), and genetic (the *CYP3A5* and *CYP3A4* gene clusters) factors significantly influence the pharmacokinetics of tacrolimus in the first 3 months post-transplantation. The application of this model, which considers the *CYP3A5* genotype, *CYP3A4* genotype, age, and BSA when selecting the tacrolimus starting dose resulted in more patients achieving target concentrations sooner than did the use of the standard bodyweight-based dosing. In a subsequent prospective, single-arm, therapeutic intervention study, the same group investigated whether determining the tacrolimus starting dose, based on a dosing algorithm that includes age, body surface area, and *CYP3A4* and *CYP3A5* genotype, leads to an increased percentage of patients achieving tacrolimus target C_0_ values on day three after transplantation, in comparison with a historic group serving as the control [43]. This dosing algorithm predicts an individual tacrolimus starting dose successfully in as many as 58% of kidney transplant patients, providing a clear improvement with regard to the control group, with 37% of patients on target in the first days after transplantation. In addition, the use of this tacrolimus dosing algorithm has the potential to reduce tacrolimus exposure outside the target concentration range. Although a randomized clinical trial is required to confirm these findings, the use of a dosing algorithm seems to minimize underexposure and overexposure to tacrolimus early after renal transplantation; therefore, it has the potential to improve graft and patient clinical outcomes.

Although there is evidence from randomized controlled clinical trials that basing the tacrolimus starting dose on the patient’s *CYP3A* genotype may be beneficial, the targeted C_0_ was achieved sooner and required fewer dose modifications when the patient *CYP3A* genotype was used [42,43,44], this has not been a universal finding in solid organ transplantation, and there is currently no convincing clinical evidence that implementation of a pharmacogenetic-based approach to tacrolimus dosing improves clinical outcomes after transplantation when tacrolimus pharmacokinetic monitoring (the analysis of the phenotype) is performed [2].

### 2.1. CYP3A5 Genotype and Tacrolimus IPV

IPV in tacrolimus exposure reflects the fluctuations in tacrolimus trough concentrations (C_0_) over a specific period of time without changes to the dose. The coefficient of variation (CV) is frequently used to calculate the C_0_ IPV (i.e., the ratio of the standard deviations of the concentrations and its mean value) in patients receiving tacrolimus [10]. Several studies have evaluated the association between tacrolimus IPV and graft outcomes regarding kidney transplantation. From these results, a high tacrolimus IPV has emerged as an important predictor of an increased risk of acute rejection, the development of a de novo donor-specific antibody (dnDSA), and an increased risk of graft dysfunction and loss [7,8,11,12,13,14]. Most of these studies considered IPV in stable patients, including patients who were assessed at least three months after the transplantation. Patients having high IPV (CV > 25–30%) show notable fluctuations in tacrolimus exposure, those values of special interest being seen in the super or sub-therapeutic range, leading to drug-adverse events (mainly nephrotoxicity) or alloreactivity and an increased risk of histological damage and rejection, respectively.

The IPV of tacrolimus C_0_ has been studied in adult and pediatric liver transplant recipients, but the evidence is more limited. The first studies in a pediatric population observed that high IPV was associated with late acute rejection related to non-adherence [45] and poor liver transplant outcomes [46]. Results from adult patients early after liver transplantation show that those patients with high IPV have an increased incidence of acute rejection, infections, and acute kidney injury (the latter probably being due to maintained tacrolimus overexposure) [47,48,49]. Interestingly, van der Veer et al. [50] studied the clinical impact of tacrolimus IPV between month 6 and month 18 after liver transplantation as a more stable time frame and found no association betweeb high IPV and immune-mediated graft injury, but a clear association was observed with a greater loss of renal function per year. Recently, Dopazo et al. [51] reported that high tacrolimus IPV (measured by the C_0_/D CV) between three and six months post-transplantation appears to be an early and independent predictor of adult patients with poorer liver transplant outcomes (worse survival rates and the loss of renal function). In addition, in liver transplant recipients, IPV leading to tacrolimus under-exposure could explain immune alloreactivity and histologic damage to the graft, reducing its metabolic activity and, therefore, tacrolimus elimination. Thus, it could eventually be the cause of tacrolimus accumulation and toxicity [52].

A systematic review from a multiorgan perspective, published by Schumacher et al. [53], concluded that high tacrolimus IPV is associated with acute rejection, dnDSA production, graft dysfunction and loss, and patient mortality in solid organ transplant recipients.

Few studies have reported that the clinical association between IPV of tacrolimus exposure and graft outcomes seems more evident in patients at high immunological risk than in those at low immunological risk. Kim et al. [54] found that a high tacrolimus IPV significantly increases the risk of graft loss after kidney transplantation, especially in patients at high immunological risk.

Given the growing body of evidence regarding poor outcomes in patients with high IPV, further study is warranted to better understand the factors influencing variability in tacrolimus exposure. The key determinant factors leading to high IPV include medication non-adherence, this being probably the single most important cause, and drug-drug interactions, which can interfere significantly with tacrolimus pharmacokinetics by the potent inhibition or induction of CYP3A5 or CYP3A4 activity, along with genetic variants [4].

Studies on the potential effects of pharmacogenetics on tacrolimus IPV are scarce. Results from two studies [55,56] show no differences in the distribution of the *CYP3A5* genotypes among renal transplant patients with a high vs. low tacrolimus IPV. Interestingly, Ro et al. [57] reported that a high tacrolimus IPV (median > 18%) is associated with kidney allograft rejection but only in those patients who are CYP3A5 expressers, whereas IPV itself is not affected by the CYP3A5 *1 allele. In agreement with this finding, Choi et al. [58] stated that pediatric kidney transplant recipients who were *CYP3A5* expressers with high tacrolimus IPV showed poor clinical outcomes, while *CYP3A5* genetic variants had no impact on tacrolimus IPV. In this pediatric cohort, high IPV was identified as a significant risk factor for the development of TCMR and dnDSA, as well as worse graft survival. Interestingly, the analysis of a large observational study conducted in kidney transplant recipients during the first six months post-transplantation showed that the IPV of tacrolimus decreases with the increase in the loss of function alleles, probably due to a greater clearance in CYP3A5*1 carriers that may favor rapid variations in tacrolimus concentrations [59]. However, currently, the influence of the *CYP3A5* genotype on tacrolimus exposure variability has not been definitively identified; further studies are required to properly evaluate the potential association between *CYP3A* genotypes and tacrolimus IPV.

### 2.2. The CYP3A5 Genotype and Tacrolimus C_0_/D Ratio

Currently, there is growing interest in identifying those patients with a fast metabolizer phenotype, who are at increased risk of poor outcomes in kidney transplantation, for the early modification of risk variables and improvement of graft and patient long-term clinical outcomes. Patients who are CYP3A5 expressers (CYP3A5*1/*3, CYP3A5*1/*1) and patients with a tacrolimus C_0_/D ratio < 1.0 ng/mL x mg may be considered fast metabolizers. In patients with a faster metabolism, the dose required to reach target tacrolimus trough concentrations is higher and, consequently, tacrolimus Cmax concentrations and tacrolimus-AUC values may be increased.

Previous studies suggest that the metabolization rate of tacrolimus, expressed as the C_0_/D ratio, would be an independent predictor of both graft and patient survival. Their results show that fast metabolizers have a high incidence of drug-related nephrotoxicity and BK-virus-associated nephropathy, whereas no significant association with TCMR or ABMR is observed [54]. Thölking et al. established several different metabolizer phenotypes, based on the tacrolimus C_0_/D ratio in kidney transplant recipients: patients with a C_0_/D ratio of <1.05 ng/mL/mg were characterized as fast metabolizers; patients with a C_0_/D ratio of 1.05–1.54 ng/mL/mg were characterized as intermediate metabolizers; those with a C_0_/D ratio of >1.55 ng/mL/mg were defined as low metabolizers [15,60].

The expression of CYP3A5 and a low C_0_/D ratio seem to be similar or interchangeable factors by which to identify fast metabolizers and predict the tacrolimus dose required to achieve target concentrations. However, significant differences may explain why the clinical impact of the C_0_/D ratio on graft and patient long-term outcomes may be more decisive than that in the CYP3A5 genotype [61]. The association between the *CYP3A5* genotype and tacrolimus exposure is robust and, on average, the CYP3A5 *1 genotype requires a higher tacrolimus dose, but a previous study showed that some patients who were CYP3A5 expressers did not have an actual fast metabolizer phenotype, and thus may reach target concentrations when receiving the conventional tacrolimus doses [21]. This fact may be partially explained by the influence of other genetic variants, such as CYP3A4*22, or drug-drug interactions that allow some patients to achieve target concentrations with standard tacrolimus doses.

Rong et al. [18] proved the significant effect of the *CYP3A5* genotype on tacrolimus C_0_/D in the first three months after kidney transplantation. It was observed that CYP3A5 expressers (*1/*1 and *1/*3) had a lower log-transformed tacrolimus C_0_/D (56% lower geometric mean) and accounted for approximately 30% of log-transformed tacrolimus C_0_/D variability during the first three months post-transplant. Interestingly, Stefanovic et al. [62] suggested that a combined assessment of tacrolimus IPV and tacrolimus C_0_/D may better identify those patients at risk of graft impairment within a three-year period following kidney transplantation. Patients with the CYP3A5 *1/*3 genotype demonstrated a low tacrolimus C_0_/D. Those patients who had either a low tacrolimus C_0_/D or high IPV during the 6–12 months after transplantation have been associated with an increased eGFR decline over a three-year period following kidney transplantation.

The initial tacrolimus dose made according to *CYP3A* genotype ensures the early achievement of target concentrations. Nevertheless, post-transplantation, other variables have to be considered for tacrolimus dose adjustment: weight, age, hematocrit, and concomitant therapeutic drugs (drug-drug interactions). For example, the use of corticosteroids in patients at higher immunological risk may lead to an increased incidence of acute TCMR or late ABMR. Steroids are known to induce CYP3A enzyme activity, wherein the tacrolimus metabolism is faster and the C_0_/D ratio becomes lower, at least for some time. This could be considered a clinical confounding factor in relation to the CYP3A5*1 genotype and its association with a high-dose tacrolimus requirement.

Taking into account the previous data, it seems that in clinical routines, the *CYP3A5* genotype may provide more accurate initial tacrolimus dose selection and a more rapid target concentration achievement but, after transplantation, the C_0_/D ratio better reflects the different factors that may result in the case of a fast metabolizer phenotype, even in those patients who are intermediate metabolizers (about 80% of Caucasians); this provides physicians with a valuable tool by which to deal with the risk factors and personal adjustment of immunosuppression.

## 3. Will Membrane Transporters Ever Become Pharmacogenetic Biomarkers of Tacrolimus Pharmacokinetics and Pharmacodynamics?

As reviewed above, drug-metabolizing enzymes and their genetic variants contribute to explaining genetic-based heterogeneity regarding tacrolimus therapeutic responsiveness and the occurrence of adverse drug reactions. However, the relative role that membrane transporters play in this regard is less clear and needs further investigation.

The so-called “rule of five” describes the minimum physicochemical requirements that a drug must accomplish to guarantee its permeability, regardless of transporters [63]. However, to what extent transporters are implicated in most pharmacokinetic- and pharmacodynamic-related events is still a matter of debate [64,65,66]. The incorporation of pre-clinical assays during drug development, aimed at predicting unwanted drug-drug interactions at the transporter level, was proposed by the International Transporter Consortium (ITC) [67] and has been developed further in recent years [68,69,70]. In this regard, the development of cell-based assays that are aimed at the characterization of drug-transporter interactions has grown rapidly so far [71]. Indeed, for any given drug, basic knowledge of influx and efflux transport mechanisms, along with robust knowledge regarding tissue and cell transporter expression profiles, is essential to better understand their pharmacokinetics, pharmacodynamics, and adverse reactions.

The pharmacogenetics of membrane transporters related to tacrolimus in solid organ transplantation has been recently reviewed by Tron et al. [6]. We refer the reader to this contribution for a detailed summary of genetic variants in transporters that are associated in some way with the use of tacrolimus in the clinical setting. However, we think that key pieces of information are lacking when dealing with tacrolimus therapy and, from a “transporter scientist” point of view, we would like to highlight where the scientific community is at present and how to proceed from now on, to answer the questions posed in this section.

### 3.1. The Role of ABC Pumps in Tacrolimus Handling

MDR1 (also known as P-glycoprotein, PgP), which is encoded by the *ABCB1* gene, has been shown to be a tacrolimus efflux transporter [72]. Although transportability was proven, the kinetic analysis of ABC proteins is not easy to perform and the functional impact of genetic *ABCB1* variants on tacrolimus transport is not yet fully understood at the molecular level. Nevertheless, from a mechanistic point of view, the *ABCB1* variants appear to be excellent candidates for determining tacrolimus pharmacokinetics and therapeutic action. MDR1 is expressed in most epithelial barriers that are known to determine tacrolimus pharmacokinetics [73,74]. MDR1 is located at the apical membrane of enterocytes, hepatocytes, and renal tubular epithelial cells, thereby determining drug absorption, as well as biliary and renal excretion. The *ABCB1* gene is highly polymorphic and several variants have been associated with tacrolimus PK/PD [6,75,76]. As recently reviewed [6], the genetic variants rs1128503 (C1236T), rs1045642 (C3435T), and rs2032582 (G2677A), which constitute a haplotype, are the most frequently reported *ABCB1* variants. Although the former two SNPs determine synonymous variants, at least in the case of C3435T, it has been unequivocally demonstrated that this SNP is associated with decreased MDR1 protein expression in the duodenum and determines increased plasma levels in orally administered digoxin, a well-characterized MDR1 substrate [77]. The molecular events behind this down-regulation of transporter expression are not clearly understood. In any case, most studies on *ABCB1*-related tacrolimus pharmacogenetics are not conclusive enough to warrant the use of *ABCB1* genotyping for PK/PD biomarker purposes [6], although this situation might be changing in the very near future.

Interestingly, the probable role of MDR1 in controlling intracellular tacrolimus levels in hepatocytes (where the drug can be excreted into bile) and lymphocytes (where the drug exerts its therapeutic action) warrants some further investigation. In this regard, two missense variants within the *ABCB1* gene (rs2032582, G2677A; rs2229109, G1199A) of liver donors, which are likely to result in reduced MDR1 function, were shown to be associated with increased intrahepatic tacrolimus levels, although no correlations with drug blood concentrations were observed [78]. Indeed, MDR1 is highly expressed in lymphocytes; in this particular case, the recipient ABCB1 variants are likely to determine intracellular tacrolimus levels and, hence, calcineurin activity [79]. The possibility that tacrolimus lymphocyte intracellular concentrations, which are likely to be related to ABCB1 genetic variants impacting MDR1 function, would become suitable pharmacodynamics biomarkers has recently been addressed by several research groups [6,79,80,81]. In particular, Tron et al. [79] have recently shown that the *ABCB1* variant rs2229109 (G1199A), which causes a serine to asparagine substitution in residue 400 that is implicated in substrate recognition, determines the intracellular accumulation of tacrolimus in PBMCs in a small cohort of liver transplant patients. This genetic polymorphism had been previously characterized at the functional level in recombinant cell lines and was shown to significantly affect intracellular tacrolimus accumulation [82]. This type of information is often lacking for most polymorphisms and highlights the need for deeper knowledge regarding the functional implications of particular genetic variants.

Previous data from our laboratory, although not related to tacrolimus pharmacodynamics but instead to the antiviral potential of raltegravir, may pave the way to better address the study of how MDR1 function impacts the immunosuppressive action of tacrolimus [83]. Low and high MDR1 function T lymphocyte subpopulations can be sorted by flow cytometry after loading them with the MDR1 substrate, Rhodamine123 (Rho123). Up to six subsets of CD4+ T cells, sorted as MDR1-low or MDR1-high cell subpopulations, can be isolated from patients. Interestingly, the percentage of MDR1 “active” cells showed strong positive correlations with the viral load of the patients in all CD4+ subsets analyzed [83]. Therefore, it might be worth determining, initially in healthy individuals, to what extent the “high” and “low” MDR-1 phenotypes are associated with particular *ABCB1* gene variants. It has been suggested that tacrolimus concentrations in PBMCs could offer a better parameter than whole-blood drug concentrations in pharmacogenetic studies [6,79]. In this regard, we have recently contributed to an expert consensus “white paper” that provides guidelines for measuring PBMC intracellular concentrations of calcineurin inhibitors [84]. The need to set up relatively easy-to-do diagnostic approaches in the clinical setting is understandable, but for informative goals, we believe that further subcellular sorting, followed by intracellular tacrolimus determinations, may be required to further elucidate how *ABCB1* pharmacogenetics impact tacrolimus action. Whether changes in MDR1 function in these CD4+ T cell subpopulations might explain inter- and intra-patient clinical heterogeneity is still an open question.

The possibility that other efflux pumps contribute to tacrolimus pharmacokinetics and pharmacodynamics has also been addressed. Genetic polymorphisms within the *ABCC2* gene, encoding multidrug-resistance protein 2 (MRP2), have recently been reviewed [6] in this regard. MRP2 tissue distribution is similar to that of MDR1. MRP2 is also found in epithelial barriers that are relevant to tacrolimus pharmacokinetics and in lymphocytes as well, but, unlike MDR1, there is no robust experimental evidence favoring the concept that MRP2 is a tacrolimus transporter.

### 3.2. Tacrolimus Influx Transporters

The role of SLC transporter proteins in tacrolimus uptake into cells is not well known. There is biochemical evidence that tacrolimus interacts with the OATP1B1 transporter [85,86]. However, the reported IC_50_ values for tacrolimus-mediated OATP1B1 inhibition are close to 4 µM [85], a concentration that is far above the reported tacrolimus Cmax [2]. On the other hand, such an interaction does not necessarily mean that the transporter is able to translocate the inhibitor. Indocyanine green, a potent OATP1B1 inhibitor, cannot be translocated by this protein [87]. OATP1B1 is encoded by the *SLCO1B1* gene [88]. This gene shows many polymorphisms and is a paradigm in the field of transporter pharmacogenetics. Indeed, OATP1B1 translocates most of the statins used to treat hypercholesterolemia; selected genetic variants within this gene are known to impact statins pharmacokinetics, some of them also being biomarkers for an increased risk of statin-associated musculoskeletal symptoms [89,90]. Importantly, guidelines to promote clinical pharmacogenetics implementation regarding *SLC01B1*-related adverse reactions to statins have been recently published [91]. OATP1B1 is a hepatocyte-specific transporter protein that is expressed at the sinusoidal (basolateral) membrane [88]. The abundance of OATP1B1 protein, but not that of other OATPs that are also expressed in the liver, such as OATP1B3 and OATP2B1, is mostly determined by genetics [92]. This supports the view that genetic heterogeneity in the *SLCO1B1* gene is a major player in the regulation of transporter expression and, accordingly, in modulating statin levels in patients [92].

OATP1B1 has been included in many pharmacogenetics studies because, as mentioned above, tacrolimus is known to interact with this transporter and inhibit its function. Nevertheless, we should keep in mind that OATP1 is liver-specific and, therefore, will not be contributing to tacrolimus absorption, nor to tacrolimus intracellular accumulation in the lymphocytes. On the other hand, it is not evident whether tacrolimus-mediated OATP1B1 inhibition is strong enough to exhibit significant drug-drug interactions in vivo. When cyclosporine A was assayed for OATP1B1 interaction, the potent inhibition of transporter function was observed, with an IC_50_ inhibitory constant of about 0.5 µM, which was one order of magnitude lower than that reported in the same study for tacrolimus [85]. Moreover, it was also reported that cyclosporine A- but not tacrolimus-induced OATP1B1 inhibition was long-lasting, both in cell lines and in primary human hepatocytes [86]. This may be relevant to the clinic setting. Potent drug-drug interactions have been reported in patients treated with cyclosporine A and statins, which in most cases can be explained by drug-drug transporter interactions [67,89]. This is not evident when looking for tacrolimus–statins interactions, although tacrolimus administration can indeed impact the pharmacokinetics of other drugs, mostly at the metabolic level, regardless of which transporters are involved [3,67,86,93]. Our current biological knowledge of this transporter protein does not support the view that OATP1B1 would be a suitable candidate to explain genetically based interpatient variability in tacrolimus PK/PD. The pharmacogenetics data available thus far do not support this possibility, although further investigation is needed.

Besides the cell-specific assays designed to pre-clinically establish drug-drug interactions (see above), for the past few years there has been increased interest in identifying endogenous transporter substrates where the blood homeostasis could be altered, following the administration of a particular transporter-competing drug (a sort of clinical probe for a particular transporter protein). Many transporters, such as OATP1B1, are highly promiscuous in terms of their substrate selectivity. A long list of endogenous OATP1B1 substrates has already been generated [88]. Among them are bile acids (cholate, taurocholate, glycocholate, tauroursodeoxycholate, and others), bilirubin and its derivatives, leukotrienes, prostaglandins, thromboxanes, thyroid hormones, and related compounds. The list is still growing. The idea is that for each particular transporter subtype, we should be able to identify an endogenous substrate that is likely to behave as an in vivo biomarker of transporter function [68]. Glycochenodeoxycholate 3-O-glucuronide (GCDCA-3G) and coproporphyrin I (CPI) have been recently identified as specific biomarkers of in vivo OATP1B1 function [94,95]. As we mentioned above, key pieces of information are lacking regarding the transporters implicated in the therapeutic action of tacrolimus. We believe that there is a need for in vivo tests of how tacrolimus administration, used as a clinical probe, alters particular transporter substrates, which could in turn help us not only to identify the key players in tacrolimus PK/PD at the transporter level but also to highlight their relevance compared to the enzymes implicated in tacrolimus metabolism. This is probably a preliminary step before exploring the pharmacogenetic side of tacrolimus action, which might involve still-unknown transporter proteins.

Lastly, it is worth mentioning that a few other membrane transporters of the *SLC* gene superfamily have been somehow related to tacrolimus pharmacokinetics [6], among them, *SLC28A3.* This gene encodes the human concentrative nucleoside transporter 3 (hCNT3). An intronic variant (rs10868152) of unknown functional consequences has been consistently associated with tacrolimus pharmacokinetics [96]. Although these authors speculated on the possibility of hCNT3 being a tacrolimus influx transporter, we do not think that this is feasible. hCNT proteins have restricted selectivity for natural nucleosides and nucleoside-derived drugs [97,98]. However, hCNT3 is a high-affinity adenosine transporter [99], while the adenosine receptors A2a have been shown to modulate MDR1 function [100]. Therefore, the possibility of indirect effects from particular SLC genetic variants on tacrolimus handling highlights the need to further expand our biological knowledge of transporter-related events that impact tacrolimus pharmacokinetics and pharmacodynamics.

## 4. Conclusions and Future Perspectives

As reviewed above, several clinical studies on transplant patients regarding the effects of genetic polymorphisms on the expression and function of drug-metabolizing enzymes and drug efflux pumps demonstrate their influence on tacrolimus drug exposure and response. Genotyping these variants has the potential to enable the individualization of the starting dose and the achievement of target blood concentrations in the first days after transplantation.

The use of dosing algorithms that incorporate *CYP3A5* and *CYP3A4* gene clusters (and probably other genes yet to come in the near future), along with clinical and demographic variants, as a way to select tacrolimus starting doses in a personalized manner might result in more patients reaching their target concentrations on day three after transplantation. This should reduce extreme exposure to tacrolimus outside the target concentration range and, consequently, would improve graft and patient outcomes. Although no convincing clinical evidence has as yet been obtained to support their implementation, there is indeed a need to develop and use dosing algorithms. They should incorporate the appropriate genotype testing strategy for each ethnic group, followed by TDM adjustments according to drug-related adverse events. Eventually, researchers and clinicians should generate robust evidence regarding algorithm suitability to achieve personalized tacrolimus therapy.

Of note, CYP3A5*3 and CYP3A4*22 genotyping may predict the tacrolimus starting dose and increase the probability of reaching target C_0_ concentrations sooner after transplantation, but, as explained above, some clinical trials failed to show clear clinical benefits to this pharmacogenetic approach on graft outcome. After transplantation, several clinical factors (drug-drug interactions, hematocrit, age, etc.) may influence the metabolism of tacrolimus. To identify those patients at risk of poor outcomes, there is a requirement to combine the tacrolimus genotype and tacrolimus phenotype (TDM). The tacrolimus IPV and the ratio of tacrolimus metabolism (C_0_/D) are recognized as risk factors that are predictive of graft and patient clinical outcomes.

Several studies, mainly in kidney transplantation and, to a limited extent, in liver transplantation, have found that IPV regarding tacrolimus exposure could predict long-term worse outcomes that are related to drug over-and under-exposure; this could help in identifying those patients that could benefit from an IPV-reducing intervention. However, further studies are required to establish standardized operational procedures regarding the method of calculation, the minimum number of C_0_ considered, the time period assessed for IPV, the IPV target, the factors influencing IPV, and even the type of primary endpoint that is associated with tacrolimus IPV (strongly associated with the time period chosen post-transplantation). Focusing on the period of time for tacrolimus C_0_ IPV analysis, the majority of the studies regarding kidney and liver transplantation have evaluated the IPV, starting at least three months after transplantation. Some authors preferred the period between the third and the sixth month post-transplantation because the patients are in a more stable situation, whereas the first weeks/months are characterized by greater fluctuation in tacrolimus C_0_ levels, due to concurrent disease and drug-drug interactions with concomitant medications, among other factors. In addition, it seems early enough to guide rapid therapeutic interventions with the aim of improving graft and patient outcomes. Another interesting point to consider is the target of the CV of tacrolimus. The recommended CV tacrolimus target is ≤20%. Results from a large number of studies show that the cutoff varies from 40% in the first month post-transplantation to below 20% after the third month post-transplantation in stable adherent patients. Of note, those recipients with a mean CV higher than 25–30% could be identified as patients at a high risk of poor outcomes. Therefore, closer therapeutic drug and clinical monitoring should be undertaken to modify the main causes leading to high tacrolimus IPV.

The clinical impact of the C_0_/D ratio on graft and patient long-term outcomes is more determinant than the CYP3A5*1 genotype because it incorporates all those conditions leading to a fast metabolizer phenotype. Moreover, the few available results on the potential effects of *CYP3A* genotypes on the IPV of tacrolimus exposure do not support a major impact of pharmacogenetics on IPV. Nevertheless, this potential association should be more properly evaluated in the context of randomized control trials with clinically relevant endpoints and by using standardized strategies for the measurement of IPV.

**Figure 1:**
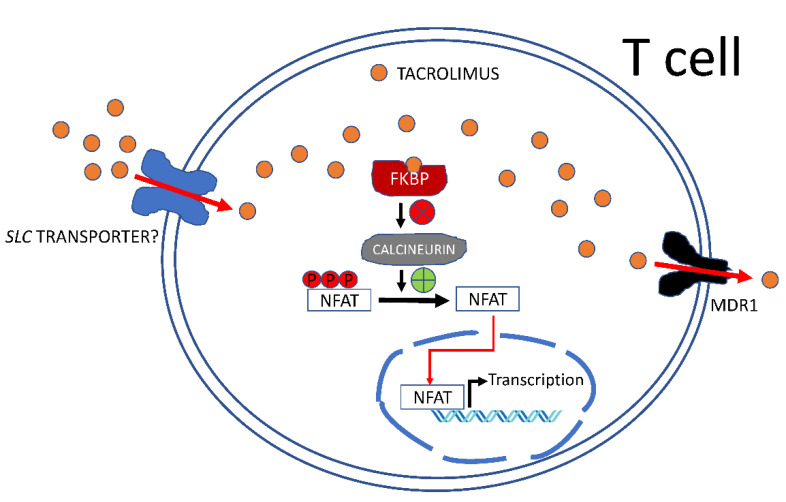
Tacrolimus handling by T cells. Tacrolimus accumulation in T cells determines the extent of calcineurin inhibition by tacrolimus-bound FK506 binding proteins (FKBP) and the subsequent inhibition of nuclear factor of activated T cell (NFAT) target genes, thereby contributing to immunosuppression. Tacrolimus intracellular concentrations are highly dependent upon the expression and functional activity of the *ABCB1* gene product, multidrug-resistance 1 (MDR1)/P-glycoprotein. Tacrolimus influx might be mediated by a still-unknown plasma membrane transporter presumably belonging to the SLC gene superfamily. The contribution of inward tacrolimus transporters to drug accumulation in T cells is not known and opens the possibility of additional gene(s) modulating its calcineurin inhibitory activity.

Beyond tacrolimus-metabolizing enzymes, it is evident that some membrane transporters also modulate tacrolimus handling and therapeutic action (Figure 1). We think that the best-characterized transporter with a clinical impact on tacrolimus therapy is the efflux pump, MDR1. Some genetic variants in its encoding gene, *ABCB1*, may affect not only tacrolimus pharmacokinetics but also intracellular drug accumulation in target cells and, consequently, calcineurin activity. Tacrolimus influx transporters, presumably belonging to the *SLC* gene superfamily, are not yet known (Figure 1). However, tacrolimus might inhibit particular transporters, some of them having clinically relevant polymorphisms (i.e., OATP1B1) that might be the basis for drug-drug interactions. Some recent data identifying other *SLC* genes as modulators of tacrolimus pharmacokinetics and pharmacodynamics need to be validated further in randomized control trials.

Last, but not least, future progress in the field will also benefit from systems biology approaches that are based upon a robust in silico analysis of genetic heterogeneity in pathways and networks, as recently developed by Zheng et al. [101].

In summary, improved therapeutic pharmacokinetic and pharmacogenetic approaches that consider patients’ clinical singularities may facilitate reaching and maintaining appropriate tacrolimus concentrations, thereby modulating the risk factors and, as a consequence, improving graft and patient long-term outcomes and survival. The task of translating this integral pharmacologic monitoring into routine clinical practice is challenging, but it is key to developing present/future individualized immunosuppressive therapies.
